# Supplementary material for: Occurrence of Chlamydiae in Corvids in Northeast Italy
Source: Animals (Basel). 2022 May 10;12(10):1226. doi: 10.3390/ani12101226 (PMC9137748; doi:10.3390/ani12101226)
Supplement: Supplementary file 1 [file animals-12-01226-s001.zip › animals-1696695-supplementary.pdf]

Table S1. *Chlamydia* species used to test the specificity of the *enoA*-based *C. psittaci* and *enoA*-based *C. abortus* rtPCRs. Origin of the strains/field isolates: [a]CHU Amiens, France; [b]INRAE Tours, France; [c]Anses Maisons-Alfort, France; [d]Faculty of Veterinary Medicine–Aristotle University of Thessaloniki, Greece; [e]Friedrich-Loeffler-Institut, Germany; [f]vaccine strain; [g]Istituto Zooprofilattico Sperimentale delle Venezie.

**Species**	**Strains/field isolates**	***C. psittaci* rtPCR**	***C. abortus* rtPCR**
C. psittaci	Loth[a], VS1[b], L2A[b], CP3[b], GR9[b], TT3[b], NJ1[b], Cal 10[b], 13-2791_BC-038[c], 13-2791_BC-065[c]	**Positive**	Negative
C. abortus	1B[b], 1H[b], 1H77[b], AB1[b], AB2[b], AB4[b], AB7[b], AB7b[b], AB13[b], iC1[b], AC1[b], POS[d], LLG[d]	Negative	**Positive**
Avian *C. abortus*	15-49D/3 (PS2)[e], 15-58D/44 (1V)[e], 15-48D/9 (PS2)[e], 15-70D/24 (PS1)[e]	Negative	**Positive**
C. pecorum	iB3[b], iB4[b], iC3[b], iC4[b]	Negative	Negative
C. felis	Dohycat[f]	Negative	Negative
C. caviae	GPIC[b]	Negative	Negative
C. trachomatis	MRC1[b]	Negative	Negative
C. gallinacea	08-1274/3[c]	Negative	Negative
C. avium	10-743/SC13[c]	Negative	Negative
C. ibidis	10-3098[c]	Negative	Negative
C. suis	21[g], MS06[g]	Negative	Negative

(A). *C. psittaci enoA* rtPCR

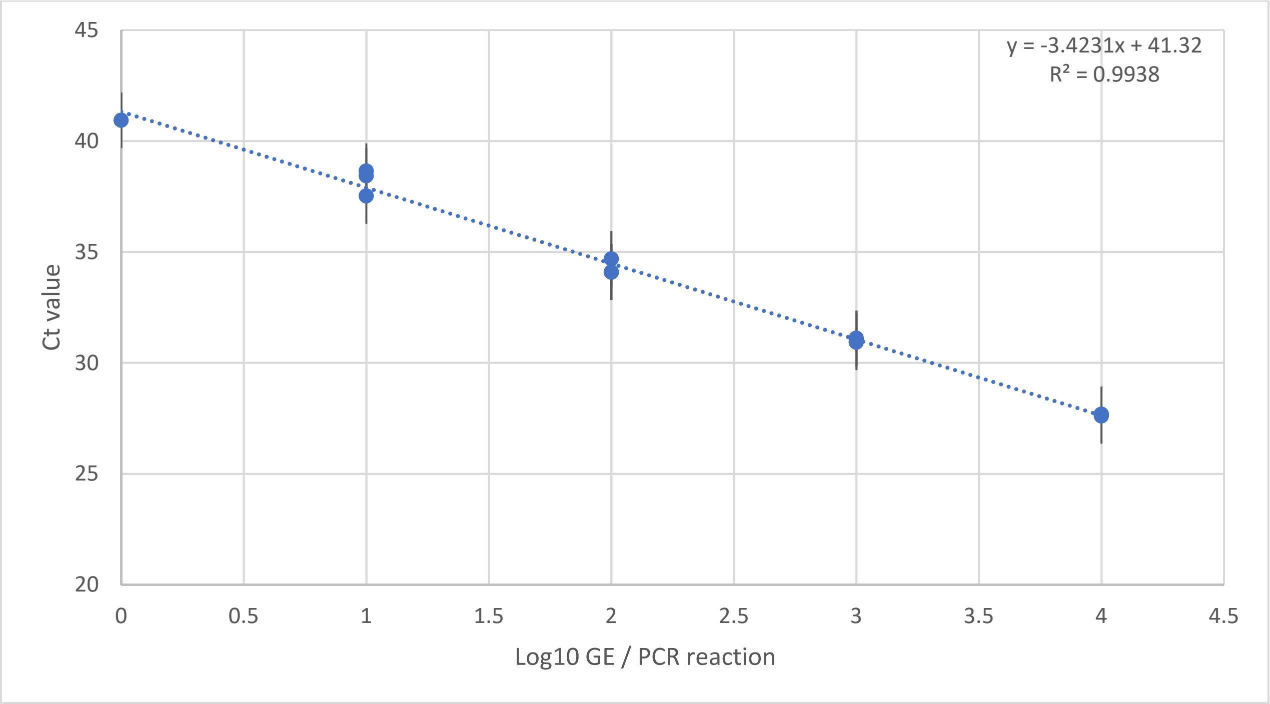

(B). *C. abortus enoA* rtPCR

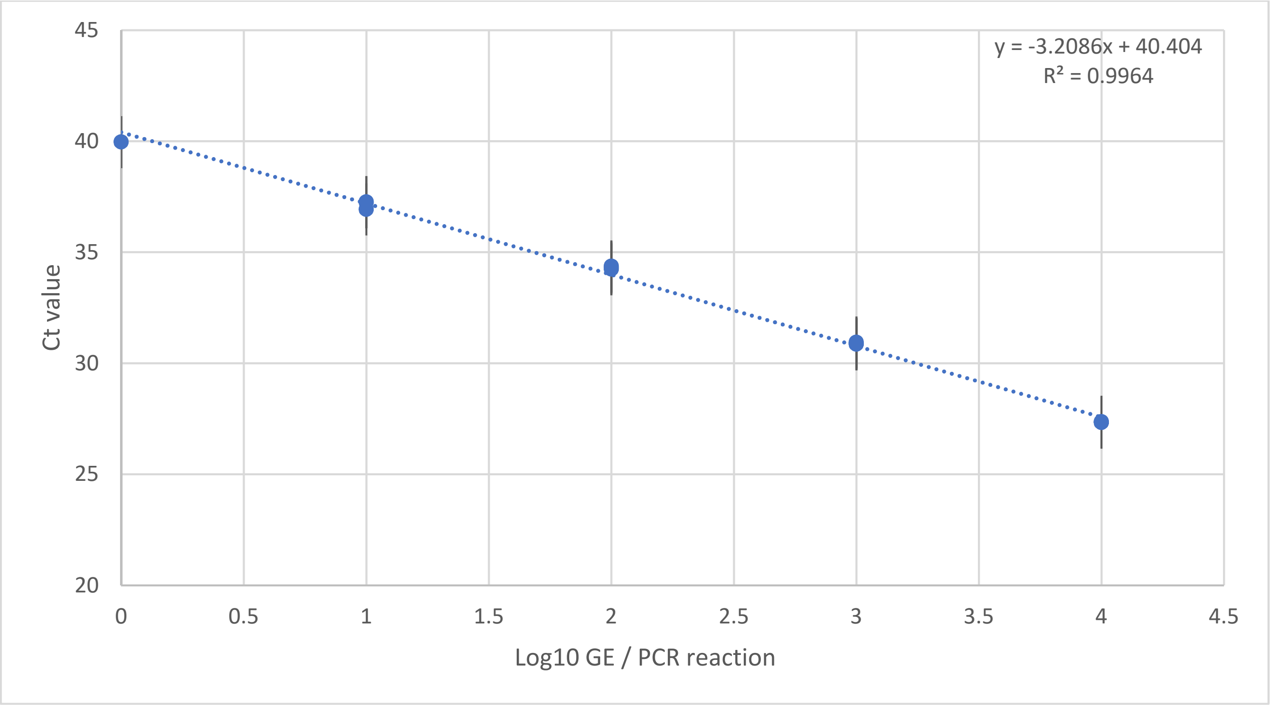

Figure S1. **Real-time PCR sensitivity test to detect the genomic DNA from *C. psittaci* Loth isolate (A) and *C. abortus* S26/3strain (B).** DNA was extracted and serially diluted. The titre of the genome equivalent (GE) was estimated from the Ct value based on the calibration curve from serial 10-fold dilutions of purified DNA extracted from cell culture containing defined numbers of GE of Loth *C. psittaci* isolate (A) and S26/3 *C. abortus* strain (B). Each dilution was subjected to real-time PCR analysis in triplicate. Y axis corresponds to Ct value and X axis corresponds to Log10 GE/PCR reaction (i.e. 2 µL). (A). *C. psittaci* parameters: the R2 linearity value from the linear regression is 0.994. y= -3.42x + 41.32 and efficiency= 95.94%%. (B). *C. abortus* parameters: the R2 linearity value from the linear regression is 0.996. y= -3.21x + 40.40 and efficiency= 104.96%.
